# Formation of Hydroxylated-Benzoyl-Benzofuranones Following the Exposure of Quercetin and Kaempferol to aNitrite-Containing Acidic Medium

**DOI:** 10.3390/molecules31132320

**Published:** 2026-07-02

**Authors:** Jocelyn Fuentes, Nélida Nina, Guillermo Schmeda-Hirschmann, Mario Aranda, Lina Patiño-Arias, Hernán Speisky

**Affiliations:** 1 Laboratory of Antioxidants, Nutrition and Food Technology Institute, University of Chile, Santiago 7830490, Chile; 2 Área de Biotecnología Microbiana, Instituto de Investigaciones Fármaco Bioquímicas, Facultad de Ciencias Farmacéuticas y Bioquímicas, Universidad Mayor de San Andrés, La Paz 0201, Bolivia; nvnina@umsa.bo; 3 Laboratorio de Química de Productos Naturales, Instituto de Química de Recursos Naturales, Campus Lircay, Universidad de Talca, Talca 3460000, Chile; schmeda.hirschmann@gmail.com; 4 Laboratory of Food & Drug Research, Faculty of Chemistry and Pharmacy, Pontificia Universidad Católica de Chile, Santiago 8320000, Chile; mario.aranda@uc.cl (M.A.); lppatino@uc.cl (L.P.-A.)

**Keywords:** quercetin, kaempferol, nitrite-derived nitrous acid, oxidation, hydroxylated-benzoyl-benzofuranones

## Abstract

Previous studies indicate that the alkali-induced oxidation of quercetin (Que) or kaempferol (Kae) converts them into hydroxylated-benzoyl-benzofuranones (BZFs), a type of metabolite whose antioxidant potency is substantially higher. To explore whether the former conversion can take place under a more biologically relevant oxidizing setting, these flavonols were incubated in a nitrite-containing acidic medium. Under such condition, the concentrations of Que or Kae rapidly decayed, simultaneously generating their corresponding BZFs. When Que and Kae were co-incubated, the depletion of the parent flavonols accelerated without changes in BZF formation. Similar results were observed when either Que or Kae were incubated with some of their corresponding 3-*O*-glycosides. In turn, when Que was co-incubated with certain flavanols or phenolic acids, the latter compounds nearly doubled not only the oxidative disappearance of Que but also its BZF formation. Interestingly, the concentrations of Que-BZF and Kae-BZF reached under all former incubation scenarios are greater than those previously reported to be needed to protect intestinal epithelial cells against the oxidative and lytic damage induced by ROS. We suggest that, if these conversions also took place in vivo, the gastric fluid could be potentially seen as a chemical milieu where the antioxidant properties of Que and Kae could be enormously amplified.

## 1. Introduction

**Figure 1:**
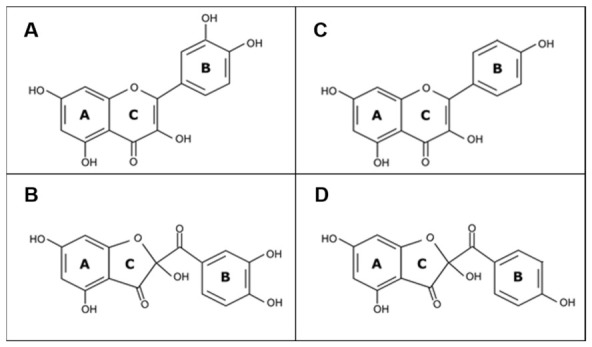
Chemical structure of quercetin (Que; part (**A**)), quercetin hydroxylated-benzoyl-benzofuranone (Que-BZF; part (**B**)), kaempferol (Kae; part (**C**)), and kaempferol hydroxylated-benzoyl-benzofuranone (Kae-BZF; part (**D**)).

Flavonoids exhibit a wide range of health-promoting bioactivities [1], the most notable of which is their ability to act as antioxidants [2]. The canonical and best-characterized mechanism of antioxidant action of flavonoids involves their capacity to interact directly with ROS, scavenging or reducing these species at the expense of their oxidation [3]. Faced with the fundamental question of whether the metabolites formed after the oxidation of a flavonoid molecule lose or retain the original antioxidant properties of the flavonoid, pioneer work conducted by Fuentes et al. [4] recently revealed that, at least in the case of quercetin (Que), a hydroxylated-benzoyl-benzofuranone (BZF) metabolite, the 2-(3,4-dihydroxybenzoyl)-2,4,6-trihydroxy-3(2H)-benzofuranone (Que-BZF; Figure 1), is formed, whose intracellular antioxidant potency is nearly 1000-fold greater than that of its precursor. This dramatic oxidation-mediated amplification of the antioxidant potency would arise from the ability of Que-BZF to activate the Nrf2-Keap 1-mediated induction of several antioxidant enzymes [5]. The latter action would account for the full protection exerted by such metabolites against the oxidative and lytic damage induced by indomethacin on Caco-2 cells, a human intestinal epithelial cell line [4], and on the gastric and intestinal mucosa of rodents [5] exposed to this ROS-generating agent. More recently, likewise observed with Que-BZF, Speisky et al. [6] reported that the hydroxylated-benzoyl-benzofuranone metabolite that results from the oxidation of kaempferol (Kae), 2-(4-hydroxybenzoyl)-2,4,6-trihydroxy-3(2H)-benzofuranone (Kae-BZF; Figure 1), is also a notably more potent antioxidant, nearly 5000-fold, than its precursor. Before uncovering the potent antioxidant properties of Que-BZF [4] and Kae-BZF [6], several investigators had previously addressed the formation and chemical characterization of these two metabolites, by exposing Que or Kae to either enzymatic, polyphenol oxidase- or tyrosinase-mediated [7,8,9], non-enzymatic, ROS-mediated [10,11], electrochemically-induced [12,13], Cu(II)-mediated [14,15], or alkaline-induced [16,17] oxidation reactions. In the case of Que, an additional and possibly biologically more relevant form of generating its corresponding hydroxylated-benzoyl-benzofuranone arose from the works of Veljovic-Jovanovic et al. [18] and Hirota et al. [19], who reported the formation of minute amounts of Que-BZF when pure Que is exposed to nitrite-derived nitrous acid under conditions that simulate the gastric fluid. Under the acidic conditions that characterize the gastric fluid milieu, nitrous acid (HONO; pKa = 3.3) is formed in situ following the protonation of nitrite (NO_2_^−^), a chemical species that continually reaches the stomach cavity through the physiological process of swallowing nitrite-containing saliva. The physiological concentration of nitrite in saliva ranges between 50 and 1000 µM (average= 200 µM) [20], depending, largely, on the contribution of dietary nitrate and the density of nitrate-reducing bacteria that, residing in the dorsal surface of the tongue, catalyze the reduction of nitrate to nitrite [21]. According to Mueller et al. [22], the concentration of nitrite in gastric juice samples obtained from young and healthy fasting volunteers ranges from 0.1–1.4 ppm (equivalent to 2.2–30 µM). In the absence of food in the stomach, the pH of the gastric fluid ranges between 1 and 2 [23]. Under such acidic conditions, nitrous acid can behave as an oxidizing, nitrosating, and/or nitrating, agent, depending on the type of phenolic substrate it interacts with. In nitrite-added HCl/KCl-containing solutions (pH 1–2), a composition that, in part, simulates the gastric fluid milieu, nitrous acid has been shown to induce the oxidation of some flavonols [18,24], the nitrosylation of several catechins and proanthocyanidins [25,26,27,28,29,30], and the nitration of certain phenolic acids [26,31,32]. The interaction between phenolic compounds and nitrous acid that takes place under an acidic environment leads not only to their oxidative conversion into diverse metabolites, but also to the reductive conversion of HONO into nitric oxide [18,26,27,33]. Considering the important roles that nitric oxide plays in human health and disease [34], most studies on the flavonoid–nitrous acid interaction have focused so far on the potential physiological implications associated with the role that the nitric oxide generated in the gastric milieu could play in the regulation of gastric mucosa formation, blood flow, and motility [33,34,35]. Comparatively, little or no attention has been given to the fact that, concomitant to the reductive conversion of nitrous acid into nitric oxide that takes place in the gastric milieu, the metabolites reported to arise from the oxidation of some flavonoids can also carry a great bioactivity potential. Within such a conceptual frame, and prompted by the recent report that Que-BZF and Kae-BZF exhibit antioxidant properties extremely higher than those of their precursor flavonols [6], in the present study, we have aimed at characterizing the potential that a nitrite-containing acidic medium, like the one present in the gastric fluid under fasting conditions, would have to serve as a chemical milieu to convert Que and Kae into their respective hydroxylated-benzoyl-benzofuranones (BZFs).

## 2. Results and Discussions

### 2.1. Chromatographic Assessment of the Formation of Hydroxylated-Benzoyl-Benzofuranones of Quercetin and Kaempferol Following Exposure of Their Precursor Flavonols to Nitrite

**Figure 2:**
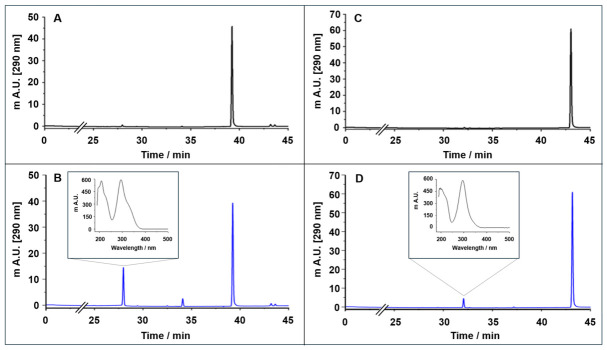
HPLC-DAD chromatograms of quercetin (**A**), quercetin (10 µM) after incubation with 20 µM of nitrite for 30 min at 37 °C (**B**), kaempferol (**C**), and kaempferol (10 µM) after incubation with 20 µM of nitrite for 30 min at 37 °C (**D**) at 290 nm. In chromatograms (**B**,**D**), the peaks corresponding to quercetin hydroxylated-benzoyl-benzofuranone and kaempferol hydroxylated-benzoyl-benzofuranone are associated with inserts containing their respective UV absorption spectra.

The presence of Que-BZF or Kae-BZF following the exposure of their precursor flavonols to nitrite was assessed in solutions obtained after incubating Que or Kae at 37 °C for 30 min in the absence or presence of nitrite (20 µM), using HPLC-DAD under the conditions described in Section 3.3. Figure 2 shows the chromatograms of Que (part A) or Kae (part C) in the absence of nitrite monitored at 290 nm, the wavelength of maximum absorption of their respective BZFs [12]. Chromatograms A and C show a single peak whose retention time is identical to that previously reported for Que (39.1 min) and Kae (43.0 min) by Speisky et al. [6]. The bottom part of Figure 2 shows the chromatograms resulting from incubating Que (part B) or Kae (part D) in the presence of nitrite. Both chromatograms show the appearance of a second peak whose retention time is 27.8 min in B and 31.9 min in D. Inserts corresponding to their respective UV absorption spectra are shown above each such peak. The retention times and the UV absorption spectra of such peaks are identical to those recently reported for the hydroxylated-benzoyl-benzofuranones of Que and of Kae generated following the autooxidation of Que and Kae in an alkaline medium [6]. Thus, the present study demonstrates that the formation of the two latter metabolites is not limited to their exposure to alkaline conditions, but that it is also observed when their precursor flavonols are exposed to a fluid that emulates, in part, the gastric environment that exist under fasting conditions; that is, acidic conditions provided by KCl/HCl at pH 2, and oxidative conditions provided by the presence of near-physiological concentrations of nitrite [22], which, in that medium, can be converted into nitrous acid [19,26]. This study considered emulating gastric chemical conditions during fasting, since in the fed state, the gastric pH temporarily rises to between 5.8 and 6.7 [23], inhibiting the formation of nitrous acid from nitrite [22]. The latter state could potentially promote the formation of BZFs, but most likely as a result of the autooxidation of Que or Kae, and not as a result of the oxidation of these flavonols by nitrous acid derived from nitrite—which is what this study sought to demonstrate. Interestingly, following the incubation of Que with nitrite (Figure 2B), in addition to Que-BZF, the formation of a third peak whose retention time is 33.3 min is observed. By chemical subtraction, this peak was chromatographically collected and analyzed by HPLC-DAD-ESI-MS/MS under the conditions described in Section 3.4. The principal ion detected has an *m*/*z* of 363, and the main fragments are *m*/*z* 271, 151, and 96 (the exact masses are shown in Figure S1). It should be noted that the here-found *m*/*z* 363 is consistent with the *m*/*z* value reported for a metabolite resulting from the oxidation of Que induced by the free radical generators 2,2-diphenyl-1-picrylhydrazyl [36] and azodiisobutyronitrile [9], or by gold chloride ions [37], having, in each of these cases, the metabolite described as a methanolic adduct of oxidized Que. However, unlike the three latter studies, in which methanol was used as the solvent for Que, in the present study methanol was not used as a solvent to dissolve this flavonol and was not part either of the mobile phase used during the chromatographic analyses. Consequently, the peak observed here at 33.3 min could not correspond to the methanolic adduct proposed by the former investigators for *m*/*z* 363. Instead of methanol, in the present study, formic acid (0.1%) was used as part of the mobile phase for all chromatographic analyses, with the aim of improving ionization efficiency in mass spectrometry and obtaining symmetrical peaks [38]. Interestingly, as reported by de Rijke et al. [39], the formation of adducts between formic acid and flavonoids rich in phenolic hydroxyl groups occurs regularly when formic acid is used in the mobile phase of the HPLC-DAD-ESI-MS/MS analysis, particularly in negative mode, similar to the one used in the present study. From a structural point of view, Que-BZF retains the same number and position of hydroxyl groups present in the A and B rings of Que [6]. Considering that no high-resolution exact-mass confirmation data is available, the here-reported 33.3 min peak should so far be seen only as a putative adduct between formic acid and the BZF of Que. Supporting the latter suggestion are the fact that adding the *m*/*z* 317 of Que-BZF to the *m*/*z* 46 of formic acid [39] yields the *m*/*z* 363 identified for the 33.3 min peak, and that the UV absorption spectrum of the 33.3 min peak is identical to the absorption maxima observed for Que-BZF (Figure S2). Regarding the major fragments at *m*/*z* 271, 151, and 96 observed for the 33.3 min peak, it should be noted that these fragments were early described by Zhou and Sadik [9] as characteristic metabolites of the final oxidative degradation of the Que molecule. Consistent with the latter, our MS/MS study also demonstrates the presence of the *m*/*z* 151 and 96 fragments for Que-BZF resulting from the oxidation of Que with nitrite (Figure S3). Therefore, the exposure of Que to nitrite in an acidic medium leads to the formation of Que-BZF, and under the analytical conditions employed here, it would also lead to the artifactual formation of an adduct between formic acid and said BZF. In the case of Kae exposed to nitrite, a peak similar to the latter peak was not detected; in fact, the chromatograms evidence only the formation of its corresponding BZF.

### 2.2. Time-Course and Concentration-Dependence Studies on the Formation of the Hydroxylated-Benzoyl-Benzofuranones of Quercetin and Kaempferol Following the Individual Incubation of Their Precursor Flavonols with Nitrite

**Figure 3:**
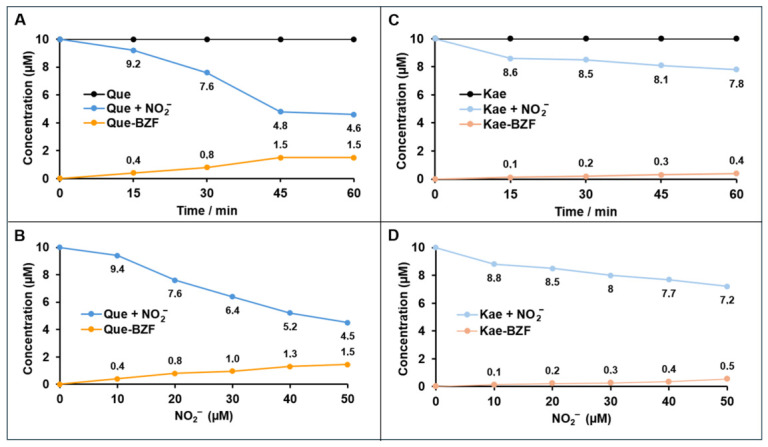
Time-course of the disappearance of quercetin (Que) and kaempferol (Kae) during their incubation (0–60 min) at pH 2 and 37 °C with 20 µM of nitrite (NO_2_^−^), and formation of their respective hydroxylated-benzoyl-benzofuranones (Que-BZF or Kae-BZF; parts (**A**,**C**), respectively). Disappearance of quercetin and kaempferol, and formation of their respective hydroxylated-benzoyl-benzofuranones, following their incubation at pH 2 during 30 min at 37 °C with increasing concentrations (10–50 µM) of nitrite (parts (**B**,**D**)). Data points plotted in this figure represent the means of at least three independent experiments, each conducted in quadruplicate. For the sake of simplicity, because the standard deviations (SD) represented less than 5% of the means, these were omitted from the figure.

Figure 3 shows the changes in the concentration of Que (part A) or Kae (part C), and in their respective BZFs when these flavonols are individually incubated (0–60 min) with a fixed concentration of nitrite (20 µM). In the absence of nitrite, no changes are observed along the incubation in the concentration of Que or Kae (each added at 10 µM). In the presence of nitrite, in contrast, Que and Kae concentrations decrease significantly during incubation. After 15 min, the concentration of Que decreases by 0.8 µM, while at 45 min, the decrease is 5.2 µM. Concurrently, at these times, the formation of Que-BZF was 0.4 µM and 1.5 µM, respectively. In the case of Kae (Figure 3C), its concentration decreases from 10 µM to 8.6 µM at 15 min and to 8.1 µM at 45 min, with negligible concentrations of Kae-BZF forming in parallel at 15 min and reaching a maximum concentration of 0.3 µM at 45 min of incubation. Thus, comparatively, Que is clearly more easily oxidized by nitrite than Kae. In line with this, using experimental conditions similar to those employed here, Takahama et al. [26] estimated that in the presence of nitrite, the rate at which Que is oxidized is six times higher than that of Kae. From a chemical-structural point of view, the only difference between Que and Kae is that the latter flavonol lacks the hydroxyl group at the C3’ position of ring B. As early reported by us in Atala et al. [40], the presence of catechol hydroxyls in ring B significantly increases the susceptibility of various flavonols to undergo oxidation. Indeed, we observed that Que, myricetin, and fisetin, all of which have a catechol hydroxyl group in ring B, were substantially more easily oxidized in an alkaline medium than Kae and morin, which lack them. Additionally, Figure 3 shows the decrease in the concentration of Que (part B) or in that of Kae (part D) after their incubation for a fixed time (30 min), but at increasing concentrations of nitrite (from 10 to 50 µM). In part B, it can be seen that as nitrite concentration is increased from 20 µM to 50 µM, the concentration of Que decreases from 7.6 µM to 4.5 µM, respectively, while the formation of Que-BZF increases from 0.8 µM to 1.5 µM. Part D shows that the concentration of Kae decreases from 8.8 µM to 7.2 µM when nitrite is increased from 20 µM to 50 µM, respectively, while the formation of Kae-BZF increases from 0.2 µM to 0.5 µM. Although these results indicate that both the disappearance of the flavonols and the formation of their respective BZFs directly depend on the nitrite concentration, in micromolar terms the magnitude of the drop in flavonol concentration induced by 50 µM of nitrite is notably greater than the magnitude of the formation of their corresponding BZFs. While in the case of Que and Kae, the decreases are 5.5 µM and 2.8 µM, in the case of their corresponding BZFs, their formation accounts for 1.5 µM and 0.5 µM, respectively. Considering such differences in magnitude, one could speculate that the nitrite-induced degradation pathway of Que or Kae may be leading to the formation of their respective BZFs, and/or that the BZFs formed are unstable, rapidly degrading into metabolites with lower molecular weights. The occurrence of both scenarios has been described following the electrochemical [9] and alkaline oxidation of Que [40]. On the other hand, the results not only confirm that Que is comparatively much more easily oxidized by nitrite than Kae, but also that the molar conversion efficiency of Que in its Que-BZF is 27%, while that of Kae in its Kae-BZF is 18%.

### 2.3. Changes in the Concentration of Quercetin and Kaempferol Hydroxylated-Benzoyl-Benzofuranones Following the Combined Incubation of Their Precursor Flavonols with Nitrite

**Figure 4:**
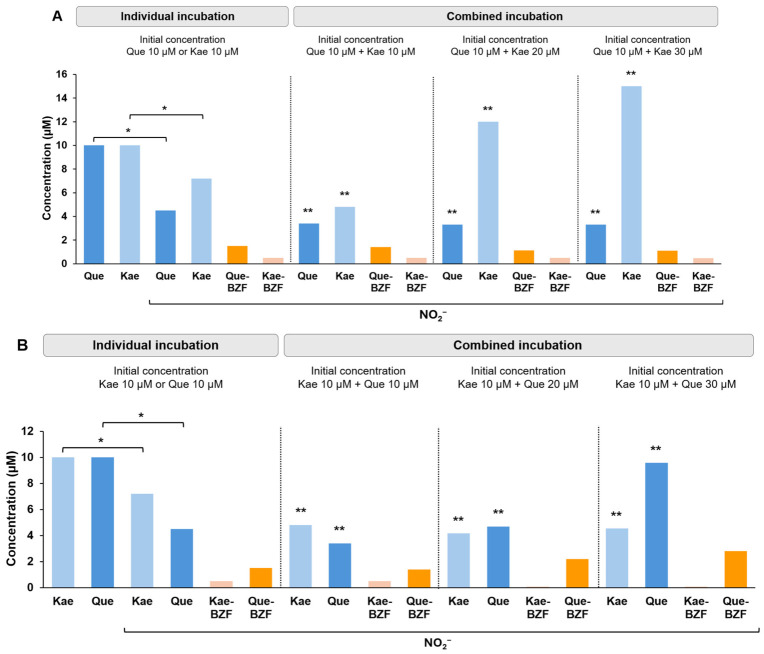
Changes in the concentration of the hydroxylated-benzoyl-benzofuranones of quercetin (Que-BZF) and kaempferol (Kae-BZF) following the individual or combined incubation of their respective precursor flavonols with 50 µM of nitrite (NO_2_^−^) during 30 min at 37 °C. (**A**) Quercetin (Que) was incubated at a fixed concentration alone (10 µM), or in the presence of increasing concentrations (10–30 µM) of kaempferol (Kae). (**B**) Kaempferol was incubated at a fixed concentration alone (10 µM), or in the presence of increasing concentrations of quercetin (10–30 µM). Significant differences: * *p* < 0.001 relative to the respective flavonol in the absence of nitrite. ** *p* < 0.001 relative to their respective flavonol after individual incubation with nitrite.

While certain foods are particularly rich in Que, it is not uncommon to find some plant foods in which Que co-occurs with Kae, as is the case with capers, almonds, and tomatoes [41]. Based on this, we wondered about the effect that the presence of one of those flavonols might have on the oxidation of the other when both are exposed to nitrite. Additionally, we evaluated the effect that the latter might have on the formation of their respective BZFs. Although the concentrations of Que and Kae resulting from their individual incubations with nitrite, and those of their respective BZFs, were already described in detail in the previous section (Figure 3B,D), the upper part of Table 1 includes these data to allow a within-the-figure comparison between that data and the concentrations of Que and Kae, and their corresponding BZFs, that result from the combined incubation of the two flavonols with nitrite, described in the bottom part of Table 1. When Que and Kae are co-incubated (37 °C for 30 min) at the same concentration (10 µM) and in combination with 10 µM nitrite, the concentrations of both flavonols drop to 5.8 µM, compared to 9.4 µM and 8.8 µM obtained, respectively, after their individual incubation. Therefore, the oxidation of each flavonol is significantly increased by the co-presence of the other. Furthermore, this oxidation is dependent on the nitrite concentration, since increasing nitrite from 10 µM to 50 µM causes the concentrations of Que and Kae to drop to 3.4 µM and 4.8 µM, respectively. From a mechanistic point of view, it is important to note that although the oxidation of Que and Kae increases when these compounds are co-incubated, such increase does not result in a comparatively greater formation of their respective BZFs. In fact, the concentration of Que-BZF and Kae-BZF obtained after the joint exposure of its precursors to 50 µM nitrite during 30 min is essentially the same as that obtained after the individual incubation of Que or Kae, that is, 1.5 µM and 0.5 µM versus 1.4 µM and 0.5 µM, respectively. In line with this, the conversion rate of Que-BZF or Kae-BZF resulting from the oxidative degradation of their precursor flavonols—incubated individually with nitrite—is significantly higher than the estimated conversion rate following their combined incubation. Therefore, it could be speculated that the intermediate metabolites formed after the combined incubation of Que and Kae follow oxidative degradation pathways distinct from those leading to BZF formation. It should be noted, however, that in the case of Que-BZF, the concentration reached is nearly three times higher than that of Kae-BZF, regardless of whether the flavonols are incubated individually or together. After chromatographic analysis of the solution resulting from the combined incubation of Que and Kae with nitrite, the peak at 33.3 min described above following the incubation of Que alone with nitrite was also observed. Therefore, the combined oxidation of Que and Kae in the presence of nitrite would result not only in the formation of their respective BZFs but also in the formation of a putative adduct between formic acid and Que-BZF. It is worth noting that the retention time at which this artifactual peak is identified in the chromatographic analysis is far from the retention time at which the formed BZFs elute; therefore, the formation of the putative BZF-formic acid adduct does not interfere with their quantification. Again, under the chromatographic conditions used in this study, no metabolites resulting from the oxidation of Kae other than its BZF were observed. Considering that after the combined incubation of Que and Kae at equimolar concentrations, the oxidation of Que is markedly increased by the presence of Kae, the question was raised as to whether this effect of Kae would be even greater upon increasing its concentration, and whether this would result in increased formation of Que-BZF and/or Kae-BZF. As shown in Figure 4A, in the presence of nitrite (50 µM), the combined incubation of Que (10 µM) with increasing concentrations of Kae (20 µM and 30 µM) results in a slightly greater, but not significant, decrease in the concentration of Que. In the case of Kae, when its concentration is increased from 10 µM to 20 µM and 30 µM it is observed that although there is greater oxidation of this flavonol, the decrease in its concentration is, in each case, close to 50% of the initial concentration added; that is, after incubating Que with 10 µM, 20 µM, and 30 µM of Kae, the concentration of the latter flavonol reaches 5.2 µM, 8.0 µM, and 15.0 µM, respectively. With regard to Que-BZF or Kae-BZF, no significant changes in the concentration of these BZFs were observed when the molar ratio of Que:Kae was increased from 1:1 to 1:3. As seen in Figure 4A, despite seeing a substantial decrease in the concentration of Kae, from 30 µM to 15 µM, the concentration of its BZF is not even slightly incremented. On the other hand, Figure 4B shows the results obtained after the combined incubation of Kae at a fixed concentration (10 µM) with increasing concentrations of Que (10–30 µM) in the presence of nitrite (50 µM). As can be seen in this figure, increasing the concentration of Que does not result in significant differences in the initial concentration of Kae. However, in the case of Que, significant decreases in its concentration are observed. That is, in the presence of Kae (10 µM), the concentration of Que decreases by 66% from 10 µM to 3.4 µM, by 77% from 20 µM to 4.7 µM, and by 68% from 30 µM to 9.6 µM. Regarding the formation of their BZFs, while the formation of Kae-BZF decreases significantly as the concentration of Que increases, from 0.5 µM (with 10 µM Que) to 0.08 µM (with 30 µM Que), that of Que-BZF increases from 1.4 µM (with 10 µM Que) to 2.8 µM (with 30 µM Que). It is worth commenting that the peak area corresponding to a putative adduct formed between formic acid and Que-BZF also increases proportionally to the concentration of Que added, that is, expressed in area units (since the molar extinction coefficient for this metabolite is not available), 41.6, 62.2, and 89.4 for 10 µM, 20 µM, and 30 µM of Que, respectively. Therefore, the increased decline in Que concentration in the presence of concentrations higher than Kae results in increased formation of metabolites resulting from its oxidation to the detriment of the formation of metabolites resulting from the oxidation of Kae. Consequently, the results show that the exposure of Que or Kae to nitrite results, depending on the time and concentration of this oxidant, in a sustained loss of the concentration of both flavonols. These changes are accompanied by a concomitant formation of their oxidized metabolites, such as Que-BZF and Kae-BZF, which was confirmed by chromatographic evidence. The linear dependence of nitrite concentration on the formation of Que-BZF observed here are in line with the findings reported by Veljovic-Jovanovic et al. [18]. Regarding Kae-BZF, there are no previous reports describing its linear formation from the oxidation of Kae with increasing concentrations of nitrite.

### 2.4. Nitrite-Induced Changes in the Concentration of Quercetin Hydroxylated-Benzoyl-Benzofuranone Following the Combined Incubation of Quercetin with Flavanols

**Figure 5:**
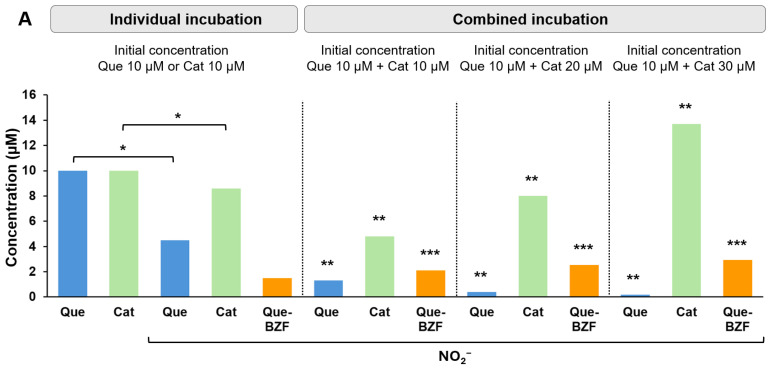

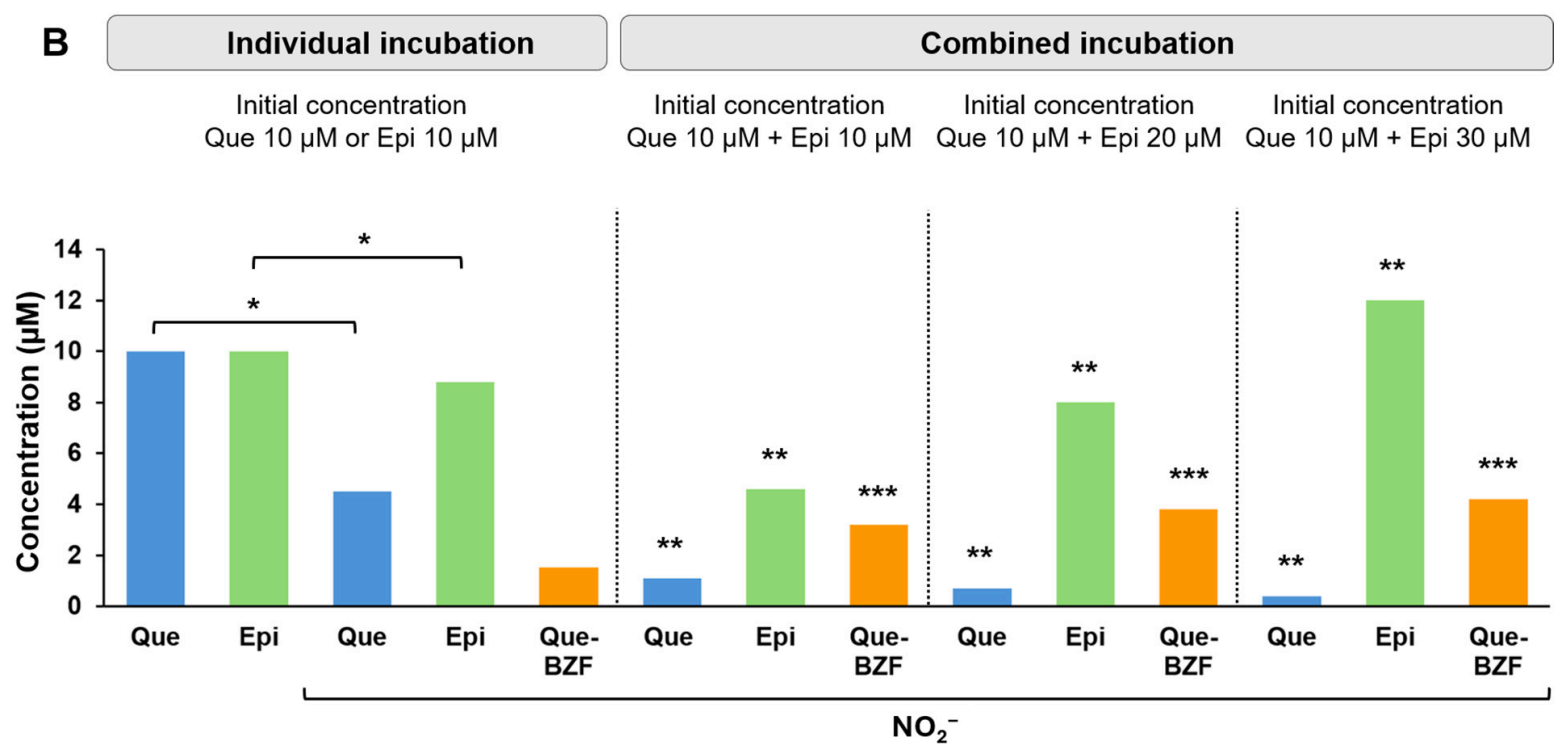
Changes in the concentration of the quercetin hydroxylated-benzoyl-benzofuranone (Que-BZF) following the combined incubation of quercetin (Que) with increasing concentrations of the flavanols catechin (Cat; part (**A**)) or epicatechin (Epi; part (**B**)) in the presence of 50 µM of nitrite. Significant differences: * *p* < 0.001 relative to the respective flavonol or flavanol in the absence of nitrite. ** *p* < 0.001 relative to their respective flavonol or flavanol after individual incubation with nitrite. *** *p* < 0.001 relative to quercetin hydroxylated-benzoyl-benzofuranone resulting from the individual incubation of quercetin with nitrite.

Results from the previous section highlight the importance that the presence of a second flavonol may have on the oxidation of a first one upon their incubation with nitrite (37 °C for 30 min), in terms of the extent of oxidation of both compounds and the formation of their respective BZFs. Unlike flavonols (such as Que and Kae), the oxidation of flavanols does not lead to the formation of BZFs. From a structural point of view, flavanols, such as catechin and epicatechin, lack the double bond between C2 and C3 and the keto group at C4, features of which are required for the formation of BZFs [42]. Given this, we wondered whether the nitrite-induced oxidation of Que and the subsequent formation of its BZF could be modified by the co-presence of catechin (Cat) or epicatechin (Epi). As shown in Figure 5A, Cat is oxidized by nitrite (50 µM) after its incubation for 30 min, dropping its concentration from 10 µM to 8.6 µM. When Cat is incubated in combination with Que, both at a concentration of 10 µM, the concentration of Cat drops from 10 µM to 4.8 µM. In turn, the concentration of Que declines from 10 µM to 1.3 µM, the latter drop being much greater than that of Que in the absence of Cat (from 10 µM to 4.5 µM). Therefore, the oxidation of both Que and Cat is enhanced by their co-presence. Moreover, the oxidation of Que (10 µM) is further exacerbated when Cat concentration is increased to 20 µM and 30 µM, yielding 0.4 µM and 0.2 µM of Que, respectively. The former increments in Que oxidation that result from the co-addition of Cat are also accompanied by increments in the formation of Que-BZF. In fact, after incubating 10 µM Que with 30 µM Cat, the concentration of Que-BZF formed in the presence of Cat (2.9 µM) is twice as much as that formed in its absence (1.5 µM). The mechanism underlying such effect of Cat remains to be established. However, Veljovic-Jovanovic et al. [18], who reported that the co-incubation of Que and Cat in the presence of nitrite leads to the formation of the 6,8-dinitrosocatechin, have suggested that a quinone intermediate of this metabolite would be capable of oxidizing Que. Therefore, if the formation of this intermediate quinone metabolite were to occur under the experimental conditions studied in this work, it could be speculated that Que might be undergoing simultaneous oxidation by nitrite and by this metabolite, which would potentially promote the increased formation of Que-BZF.

Regarding epicatechin, we observed that this flavanol is also oxidized by nitrite (Figure 5B), with its concentration decreasing from 10 µM to 8.8 µM. When Epi (10 µM) was incubated together with Que (10 µM), such decrease is exacerbated, lowering its concentration to 4.6 µM. In the case of Que, the decrease from 10 µM to 4.5 µM observed after the individual incubation is drastically greater in the presence of Epi, dropping its concentration from 10 µM to 1.1 µM. The latter effect of Epi is further exacerbated when its concentration is raised to 20 µM and 30 µM, resulting in a final concentration of 0.7 µM and 0.4 µM of Que, respectively. Regarding Que-BZF, the presence of Epi also significantly promotes its formation, increasing its concentration from 1.5 µM, seen in the absence of Epi, to 4.2 µM seen in the presence (30 µM). As in the case of Cat, a 6,8-dinitrosoepicatechin metabolite has also been reported following the oxidation of Epi with nitrite [43].

### 2.5. Nitrite-Induced Changes in the Concentration of Quercetin Hydroxylated-Benzoyl-Benzofuranone Following the Combined Incubation of Quercetin with Phenolic Acids

In this section we investigated whether, as previously seen with flavanols, the interaction between Que and non-flavonoid compounds, like gallic acid (GA) or protocatechuic acid (PA), can also favor the oxidation of Que and the formation of Que-BZF. As shown in Figure S4, after its exposure to nitrite (50 µM), the concentration of Que, which in the absence of GA drops from 10 µM to 4.5 µM, was significantly increased in the presence of GA (10 µM), falling from 10 µM to 0.6 µM. The greater oxidation of Que associated with the presence of GA translates also into a greater formation of Que-BZF. In fact, Que-BZF concentration increases as GA concentration is increased from 10 µM to 20 µM and 30 µM, reaching 2.2 µM, 2.8 µM, and 3.5 µM, respectively. In the case of the Que-PA co-incubation, the drop in the concentration of Que is considerably higher in the presence of PA compared to the absence of PA, the drop being from 10 µM to 1.7 µM versus to 4.5 µM, respectively (Figure S5). Regarding the changes in Que-BZF, as observed with GA, its formation also increases significantly in the presence of PA, and as PA concentration is increased from 10 µM to 20 µM and 30 µM, the concentration of Que-BZF increased from 2.1 to 2.5, and to 2.9 µM, respectively.

### 2.6. Nitrite-Induced Changes in the Concentration of Quercetin or Kaempferol Hydroxylated-Benzoyl-Benzofuranones Following the Combined Incubation of Quercetin or Kaempferol with Their Respective 3-O-Glycosides

It is common to find that flavonols, such as Que and Kae, co-occur in foods in their aglycone form alongside their glycosylated structures [41]. In view of this, we explored the potential effect that increasing concentrations of 3-*O*-glycosides of Que or Kae might have on the oxidation of their aglycones, and on the formation of the Que-BZF and Kae-BZF. In the case of Que, this flavonol was incubated in combination with isoquercitrin (ISO), rutin (RUT), or hyperoside (HYP), while in that of Kae, the co-incubation was with nicotiflorin (NIC) or with astragalin (AST), all in the presence of nitrite. These studies allowed us to evaluate, for first time, the effects of exposing the 3-*O*-glycosides ISO, HYP, NIC, and AST to nitrite following their individual or combined incubation with Que or with Kae. The co-incubation of aglycone flavonols with their glycosides in the presence of nitrite in an acidic medium takes on physiological significance when one considers that, following mastication of different edible plants, gastric fluid always receives a mixture of both structural forms [24].

**Figure 6:**
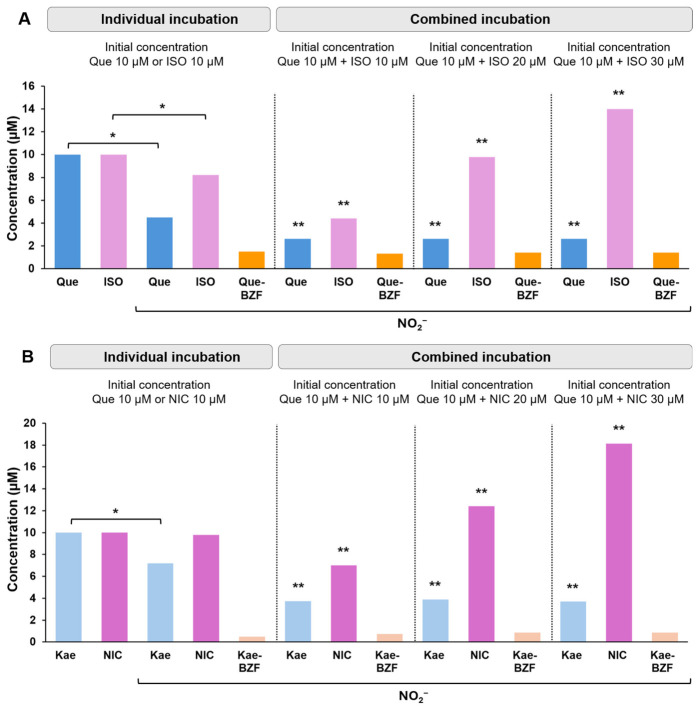
Changes in the concentration of the quercetin hydroxylated-benzoyl-benzofuranone (Que-BZF) following the incubation of their flavonol precursors with 50 µM nitrite in the presence of increasing concentrations of 3-*O*-glycosylated of flavonols. (**A**) Quercetin (Que) was incubated in the presence of increasing concentrations of isoquercitrin (ISO). (**B**) Kaempferol was incubated in the presence of increasing concentrations of nicotiflorin (NIC). * *p* < 0.001 relative to the respective flavonol in its aglycone form or in its glycosylated form in the absence of nitrite. ** *p* < 0.001 relative to the respective flavonol in its aglycone form or in its glycosylated form after individual incubation with nitrite.

From a structural point of view, the only difference between ISO and Que is that the former molecule has a glucopyranoside group instead of a hydroxyl group at the C3 position [44]. Although the latter structural feature precludes ISO from being converted into its BZF, it does not prevent ISO from undergoing oxidation by nitrite (50 µM); in fact, as shown in Figure 6A, its sole exposure to nitrite leads its concentration to decrease from 10 µM to 8.2 µM. Under identical incubation conditions, Que exhibits a substantially greater decrease, from 10 µM to 4.5 µM. The smaller decrease in ISO concentration may be due to the fact that blocking the C3 position of ring C with the glucopyranoside group would affect the stabilization of the phenoxyl radicals formed in the B ring, thus limiting their conversion of such radicals into stable quinones [40]. On the other hand, following the combined incubation of ISO and Que, a more pronounced decrease in the concentrations of both flavonols is observed: while the concentration of ISO drops from 10 µM to 4.4 µM, that of Que drops from 10 µM to 2.6 µM. Interestingly, under the experimental conditions of the present study, the greater decrease in Que concentration induced by nitrite seems to be maximal at a 10 µM or lower concentration of ISO, as no further decreases in Que concentration were observed after raising ISO concentration to 20 µM or 30 µM. It should be noted, however, that the maximal decrease in Que concentration associated with the addition of ISO takes place under conditions where ISO oxidation increases in direct proportion to the magnitude its concentration is increased. Regarding Que-BZF formation, unlike what was observed after the combined incubation of Que with flavanols or phenolic acids, the incubation of Que with ISO at equimolar concentrations (10 µM) does not result in an increased formation of Que-BZF. Indeed, as shown in Figure 6A, after 30 min of incubation, a 1.3 µM Q concentration of Que-BZF is formed. The latter value is lower than that arising from the sole exposure of Que to nitrite (1.5 µM), but the observed difference is not statistically different. Increasing the concentration of ISO to 20 µM or 30 µM had no effect on Que-BZF formation (1.4 µM). Therefore, although the addition of ISO to Que exacerbates the nitrite-induced oxidative decline in the aglycone concentration, such effect does not translate into a further increase of Que-BZF formation. The possibility that part of the Que-BZF formed during the incubation of Que with ISO may have degraded seems to be unlikely since HPLC-DAD analysis of samples obtained from such incubation reveals the absence of the peaks of the metabolites formerly described to be formed after the oxidative degradation of Que-BZF, like trihydroxy-benzoic acid or protocatechuic acid [9]. On the other hand, as shown in Figures S6 and S7, the present study shows that like ISO, the two other tested 3-*O*-glycosides, RUT and HYP, are oxidizable by nitrite (50 µM), with their concentrations decreasing from 10 µM to 8.2 µM and 8.3 µM, respectively. Following the combined incubation of equimolar concentrations (10 µM) of Que and RUT, or Que and HYP, the decrease in the initial concentration of the aglycone is, in both cases, from 10 µM to 2.5 µM. Such decline is greater than that seen after individual incubation of Que, which is from 10 µM to 4.5 µM. In the case of the 3-*O*-glycosides RUT and HYP, the concentration of both drops from 10 µM to 4.4 µM, and from 10 µM to 4.3 µM, respectively. Concurrently, as observed with ISO, the greater decrease in the concentration of Que is not associated with greater formation of Que-BZF, even when the concentration of RUT or HYP is increased to 20 µM or 30 µM.

The 3-*O*-glycoside of Kae, NIC, possesses a rutinoside group bound at the C3 position of its C ring instead of the hydroxyl group found in Kae. Figure 6B shows that, in the presence of nitrite, NIC slightly drops its concentration, from 10 μM to 9.8 μM. This result is to be expected considering that NIC, in addition to having the C3 position blocked, exhibits, like Kae, only one hydroxyl group in its B ring; the latter feature would imply a very limited capacity to allow electron resonance, displaying comparatively a much lower reactivity toward oxidants [40]. The drop in NIC concentration is, however, exacerbated in the presence of Kae (10 µM), as it falls from 10 µM to 7.0 µM. In parallel, the addition of NIC to the incubation leads to a greater oxidation of Kae, decreasing the concentration of the latter to 3.7 µM, which is compared with the 7.2 µM concentration observed in the absence of NIC. Regarding Kae-BZF, it should be noted that the greater decrease in Kae concentration seen in the presence of NIC does not result in increased formation of Kae-BZF. In fact, the concentration of the latter is only slightly higher (0.7 µM) and statistically, it is not significantly different from that seen in its absence (0.5 µM). On the other hand, in the case of AST, the here-tested second 3-*O*-glycoside of Kae, Figure S8 shows that its concentration is slightly reduced in the presence of nitrite, dropping from 10 µM to 9.6 µM. Following the co-incubation of Kae with AST, it is observed that, as previously seen with NIC, the concentrations of both the aglycone and the glycoside fall considerably more than when each of them is singly exposed to nitrite, that is, from 10 µM to 7.2 µM for AST, and from 10 µM to 3.8 µM for Kae, values that should be compared with a 7.2 µM concentration obtained when Kae is incubated in the absence of AST. Regarding Kae-BZF, it can be seen that the glycoside-induced greater decrease in Kae concentration does not result in a significantly greater formation of Kae-BZF, as the concentration of the latter remains constant at 0.8 µM even after incubating Kae with 20 µM or 30 µM AST (see Figure S8). The fact that the 3-*O*-glycosides of Que and Kae do exacerbate the oxidation of Que and Kae, but not their conversion into their corresponding BZFs, is not in line with what is seen following the incubation of Que or Kae with various flavanols and phenolic acids. This apparently paradoxical behavior could be explained, hypothetically, as follows: while nitrite-derived nitrous acid is able to equally induce the oxidation of the hydroxyl groups of the B ring of the aglycones as well as those present in the glycosides, the thus-formed semi-quinones and quinones intermediates differ in terms of their metabolic fate. In the case of the aglycones, in which a non-phenolic hydroxyl group is bound to the C3 position of their C rings, the semi-quinones and quinones formed undergo transformations that finally lead to the formation of their corresponding BZFs [12]. In turn, in the case of the semi-quinones and quinones derived from the 3-*O*-glycosides, in which a sugar is bound to the C3 position of their C rings, the possibility that these species form BZFs is structurally precluded. We hypothesize that the latter reactive species would selectively react with the hydroxyl groups of ring A of the Que or Kae aglycones, inducing an oxidation that exacerbates their disappearance but that does not lead to furthering the formation of their BZFs.

## 3. Materials and Methods

### 3.1. Chemicals

All polyphenols used in this study were purchased from Sigma-Aldrich (St. Louis, MO, USA), and their purity was as follows: quercetin (≥95%), kaempferol (≥98%), (+)-catechin hydrate (≥98%), (−)-epicatechin (≥90%), gallic acid (≥98%), protocatechuic acid (≥98%), isoquercitrin (≥95%), rutin (≥94%), hyperoside (≥99%), nicotiflorin (≥98%), astragalin (≥99%). Acetonitrile (ACN) and formic acid were HPLC grade. Ethanol, sodium acetate, sodium nitrite, sodium chloride, and hydrochloric acid were analytical grade and purchased from Merck (Darmstadt, Germany).

### 3.2. Incubation Conditions

The incubation medium was prepared, essentially, as previously described by Takahama et al. [26]. In all individual or combined incubations, the incubation media contained 100 µL of Que and/or Kae (10–30 μM), 350 µL of KCl/HCl 25 mM (pH 2), and 50 µL of sodium nitrite (10–50 μM). The flavonols were initially dissolved in pure ethanol to ensure complete dissolution. The stock solution of each flavonol was prepared immediately before each incubation, after which it was discarded. Intermediate dilutions of each flavonol were prepared using a mixture of ethanol/ultrapure distilled water (50/50 *v*/*v*) to ensure the compound’s solubility in the incubation mixture. The incubations were carried out in a temperature-controlled bath at 37 °C, and were kept under constant shaking (80 rpm) during 0–60 min. Reactions were ended by neutralizing the incubation mixtures by the addition of sodium acetate buffer (250 mM, pH 7). Identical incubation conditions were employed in experiments in which Que or Kae were co-incubated with either the flavanols catechin or epicatechin, the phenolic acids gallic or protocatechuic, or the 3-*O*-glycosides isoquercitrin, rutin, hyperoside, nicotiflorin, or astragalin. As done with Que and Kae, the 3-*O*-glycosides were dissolved initially in ethanol. The flavanols and the phenolic acids were dissolved in ultrapure distilled water.

### 3.3. HPLC-DAD Analysis of the Incubation Mixtures

In order to assay the concentrations of Que or Kae, and/or those of their corresponding BZFs, samples taken at different times of incubation were subjected to HPLC-DAD analysis using an Agilent 1200 series pump (Santa Clara, CA, USA), equipped with an autosampler and a photodiode array detector (DAD) (Santa Clara, CA, USA). The HPLC system was controlled by Agilent ChemStation (Agilent Technologies 2010, Santa Clara, CA, USA). The chromatographic conditions used in this study are identical to those previously reported by us [6]. The mobile phase consisted of a mixture of (A) acetonitrile and (B) 0.1% aqueous formic acid, whose composition was varied by employing the following HPLC gradient program: 0–15.0 min 10% A, and 15.0–50.0 min 10–60% A, and returned to starting conditions in the following 10 min. The column used was a 250 × 4.6 mm, i.e., 5 µm, Kromasil 100-5-C18 (AkzoNobel, Bohus, Sweden). Other chromatographic conditions were as follows: sample injection volume of 100 μL, flow rate of 0.8 mL/min, and oven column set at 25 °C. The absorbance of the eluates was monitored at 370 nm for Que or Kae, and at 290 nm for Que-BZF or Kae-BZF. The concentrations of the flavonols and their respective BZFs were estimated from the areas under the curves of their chromatographic peaks using standard curves prepared just before the experiments. The standard deviation of each determination was always less than 5%. The Que-BZF and Kae-BZF calibration curves showed excellent linearity in the concentration range of 0.01–10 µM, both with a coefficient of determination R^2^ = 0.9998. The calculated limit of detection (LOD) and limit of quantification (LOQ) were 0.001 µM and 0.01 µM, respectively. Identical chromatographic conditions were used in monitoring the concentrations of the formerly referred flavanols, phenolic acids, and 3-*O*-glycosides.

### 3.4. ESI-MS/MS Analysis of the Metabolites Resulting from the Nitrite-Induced Oxidation of Quercetin and Kaempferol

To confirm the chemical identity of the HPLC-DAD peaks associated with the BZFs Que-BZF and Kae-BZF, and to assess that of the peak that elutes at 33.3 min (as a putative metabolite of Que oxidation), samples of each of such peaks were collected using an Agilent 1260 Infinity automated fraction collector (coupled to the HPLC system), dried immediately after at 23 °C under a nitrogen flow, and analyzed by HPLC-DAD-ESI-MS/MS. To establish the chemical innocuousness of the drying process, specifically the absence of new peaks, each dried fraction was reconstituted in a mixture of 0.1% (*v*/*v*) formic acid in MS-grade water and 0.1% (*v*/*v*) formic acid in MS-grade acetonitrile (50/50 *v*/*v*), and analyzed using a Waters UPLC system (Milford, MA, USA) equipped with a Quaternary Solvent Manager-R pump, a Sample Manager FTN-R autosampler, a CH-30A column heater, a GL Sciences (Shinjuku-ku, Tokyo, Japan) 2.6 µm InertCore Plus C18 column (100 × 4.6 mm, UP) set at 30 °C, and a DAD at 370 nm and 290 nm. The mobile phase consisted of a mixture of (A) 0.1% (*v*/*v*) formic acid in MS-grade water, and (B) 0.1% (*v*/*v*) formic acid in MS-grade acetonitrile. Samples were eluted using the following gradient at a flow rate of 0.4 mL/min: 0–2 min 15.0% B, 2–6 min 15–30% B, 6–10 min 30–50% B, 10–14 min 50–70% B, 14–15 min 70–80% B, 15–18 min 80–15% B, and returned to starting conditions in the following 2 min. Subsequently, the ESI-MS/MS spectra were acquired using a Waters SYNAPT XS mass spectrometer (Waters Corp., Milford, MA, USA) equipped with electrospray ionization (ESI). Acquisition: run time 0–20 min, ESI (-), resolution analysis mode, normal dynamic range; Tune settings: capillary voltage of 2.0 kV, sampling cone 30 V, source offset 4 V, source 120 °C, desolvation 400 °C, cone gas 50 L/h, desolvation gas 500 °C, nebulizer 6.0 bar; TOF MS: scan range (50–1500 Da), scan time 0.3 s, continuum data format; Collision energy: Low trap collision 4 eV, high transfer collision 15–45 eV; Analysis: MS spectra were collected by MassLynx Software Data Acquisition (Version 4.2, Waters Corp., Milford, MA, USA).

### 3.5. Statistical Analysis

The data shown in each of the figures and in the table represent the means of at least three independent experiments, each conducted in quadruplicate. In all cases, the standard deviation was less than 5% of the means; therefore, it was decided to omit them from the figures and table. Data were analyzed using GraphPad Prism 5 statistical software (La Jolla, CA, USA). Statistical significance of the differences between the experimental conditions was assessed with the analysis of variance (ANOVA) and post hoc Bonferroni test, as appropriate.

## 4. Conclusions and Potential Implications

This in vitro chemical simulation study not only demonstrates nitrite-derived nitrous acid ability to induce, in a concentration- and time-dependent manner, the oxidative degradation of the flavonols Que and Kae when these are present in an acidic environment that resembles gastric fluid under fasting conditions, but also reveals that their corresponding BZFs are formed concurrently with their degradation. Although these changes already occur when these flavonols are individually exposed to nitrite, their co-incubation exacerbates the degradation of both flavonols, but it does not further the formation of their corresponding BZFs. Similar results are observed when either Que or Kae are incubated with some of their corresponding 3-*O*-glycosides. However, it is worth noting that the concentrations of Que-BZF and Kae-BZF (reaching 100–1400 nM) after either the individual or the combined oxidation of low concentrations (10–30 µM) of their precursors are absolutely relevant to those reported to be required to protect human intestinal epithelial cells against the oxidative and cellular damage induced by ROS [4,6]. Interestingly, when Que is co-incubated with certain flavanols or phenolic acids that, due to their structural characteristics, are unable to form BZFs [42], these nearly doubled not only the oxidative disappearance of Que but also the formation of its BZF. If these oxidative conversions could actually take place also in the gastric environment in vivo, one might speculate that an increased formation of Que-BZF and Kae-BZF could be expected to occur following the ingestion of foods that, being rich in Que or Kae aglycones, also contain non-flavonol phenols such as mentioned above. The mechanism underlying the increase in BZF formation resulting from the combined exposure of Que and Kae to nitrite remains to be established; however, it is highly likely that the interaction between these flavonols involves a mechanism of oxidative synergy. Indeed, according to Takahama and Hirota [22], the co-exposure of Que and various phenols to nitrite would exacerbate the oxidation of the flavonol as a result of intermediate metabolites with pro-oxidant properties (such as semi-quinones or quinones), generated during the oxidation of the other phenols [45], promoting the oxidation of Que together with nitrite. In terms of the potential impact of the results reported here, it is worth noting that the oxidative conversion of Que and Kae into their respective BZFs, shown here to occur in a nitrite-containing acidic medium, should not be viewed as a loss of the antioxidant properties of these flavonols, but rather as a process that could result in the amplification of the antioxidant properties of the fluid containing them. To support this, however, and eventually extrapolate the results of the present chemical conversion study into a somehow more physiological one, it would be necessary to conduct experiments involving the evaluation of the antioxidant and cytoprotective properties of fluids containing BZFs in cellular models of gastric and intestinal epithelium exposed to ROS.

## Figures and Tables

**Table 1:** Changes in the concentration of quercetin (Que) and kaempferol (Kae) following their individual or combined incubation with increasing concentrations of nitrite (10–50 µM), and in the concentration of their corresponding hydroxylated-benzoyl-benzofuranones (Que-BZF or Kae-BZF). For Que-BZF and Kae-BZF, the values in parentheses accompanying each reported concentration correspond to the estimated conversion percentage, calculated as the concentration of BZF formed/concentration lost from its respective flavonol precursor × 100.

	NO_2_^−^	0	10	20	50	µM
Individual incubation	Que	10	9.4 *	7.6 *	4.5 *	µM
	Kae	10	8.8 *	8.5 *	7.2 *	
	Que-BZF	0	0.4 (66.7%)	0.8 (33.3%)	1.5 (27.3%)	
	Kae-BZF	0	0.1 (8.3%)	0.2 (13.3%)	0.5 (17.9%)	
Combined incubation	Que (+Kae 10 µM)	10	5.8 **	5.0 **	3.4 **	
	Kae (+Que 10 µM)	10	5.8 **	5.2 **	4.8 **	
	Que-BZF	0	0.4 (9.5%)	0.7 (14.0%)	1.4 (21.2%)	
	Kae-BZF	0	0.1 (2.4%)	0.2 (4.2%)	0.5 (9.6%)	

Data points plotted in this table represent the means of at least three independent experiments, each conducted in quadruplicate. For the sake of simplicity, because the SD represented less than 5% of the means, these were omitted from the figure. Significant differences: * *p* < 0.001 relative to the respective flavonol in the absence of nitrite. ** *p* < 0.001 relative to their respective flavonol after combined incubation in the absence of nitrite.

## Data Availability

The original contributions presented in this study are included in the article. Further inquiries can be directed to the corresponding author.

## Supplementary Materials

The following supporting information can be downloaded at: https://www.mdpi.com/article/10.3390/molecules31132320/s1, Figure S1. ESI-MS/MS chromatograms of the adduct formed between formic acid and quercetin hydroxylated-benzoyl-benzofuranone. Primary (A) and secondary (B) fragmentation. Figure S2. HPLC-DAD chromatograms of quercetin in the presence of nitrite. The peak corresponding to adduct formed between formic acid and quercetin hydroxylated-benzoyl-benzofuranone is associated with insert containing their respective UV absorption spectra. Figure S3. ESI-MS/MS chromatogram of quercetin hydroxylated-benzoyl-benzofuranone. Primary (A) and secondary (B) fragmentation. Figure S4. Changes in the concentration of the quercetin hydroxylated-benzoyl-benzofuranone following the incubation of quercetin with nitrite in the presence of increasing concentrations of gallic acid (GA). Significant differences: * *p* < 0.001 relative to the respective flavonol or phenolic acid in the absence of nitrite. ** *p* < 0.001 relative to their respective flavonol or phenolic acid after individual incubation with nitrite. *** *p* < 0.001 relative to quercetin hydroxylated-benzoyl-benzofuranone resulting from the individual incubation of quercetin with nitrite. Figure S5. Changes in the concentration of the quercetin hydroxylated-benzoyl-benzofuranone following the incubation of quercetin with nitrite in the presence of increasing concentrations of protocatechuic acid (PA). * *p* < 0.001 relative to the respective flavonol or phenolic acid in the absence of nitrite. ** *p* < 0.001 relative to their respective flavonol or phenolic acid after individual incubation with nitrite. *** *p* < 0.001 relative to quercetin hydroxylated-benzoyl-benzofuranone resulting from the individual incubation of quercetin with nitrite. Figure S6. Changes in the concentration of the quercetin hydroxylated-benzoyl-benzofuranone following the incubation of quercetin with nitrite in the presence of increasing concentrations of their 3-*O*-glycosylated rutin (RUT). * *p* < 0.001 relative to the respective flavonol in its aglycone form or in its glycosylated form in the absence of nitrite. ** *p* < 0.001 relative to the respective flavonol in its aglycone form or in its glycosylated form after individual incubation with nitrite. Figure S7. Changes in the concentration of the quercetin hydroxylated-benzoyl-benzofuranone following the incubation of quercetin with nitrite in the presence of increasing concentrations of their 3-*O*-glycosylated hyperoside (HYP). * *p* < 0.001 relative to the respective flavonol in its aglycone form or in its glycosylated form in the absence of nitrite. ** *p* < 0.001 relative to the respective flavonol in its aglycone form or in its glycosylated form after individual incubation with nitrite. Figure S8. Changes in the concentration of the kaempferol hydroxylated-benzoyl-benzofuranone following the incubation of kaempferol with nitrite in the presence of increasing concentrations of their 3-*O*-glycosylated astragalin (AST). * *p* < 0.001 relative to the respective flavonol in its aglycone form or in its glycosylated form in the absence of nitrite. ** *p* < 0.001 relative to the respective flavonol in its aglycone form or in its glycosylated form after individual incubation with nitrite.

## Abbreviations

The following abbreviations are used in this manuscript:

AST	Astragalin
BZF	Hydroxylated-benzoyl-benzofuranone
Cat	catechin
Epi	epicatechin
GA	gallic acid
HONO	nitrous acid
HYP	hyperoside
ISO	isoquercitrin
Kae	kaempferol
Kae-BZF	2-(4-hydroxybenzoyl)-2,4,6-trihydroxy-3(2H)-benzofuranone
Keap 1	Kelch ECH associating protein 1
NIC	nicotiflorin
NRF2	Nuclear factor erythroid 2-related factor 2
NO_2_^−^	nitrite
PA	protocatechuic acid
Que	quercetin
Que-BZF	2-(3,4-dihydroxybenzoyl)-2,4,6-trihydroxy-3(2H)-benzofuranone
ROS	reactive oxygen species
RUT	rutin
SD	standard deviation
