# Supplementary figures and images for: Formation of Hydroxylated-Benzoyl-Benzofuranones Following the Exposure of Quercetin and Kaempferol to aNitrite-Containing Acidic Medium
Source: Molecules. 2026 Jul 2;31(13):2320. doi: 10.3390/molecules31132320 (PMC13363257; doi:10.3390/molecules31132320)

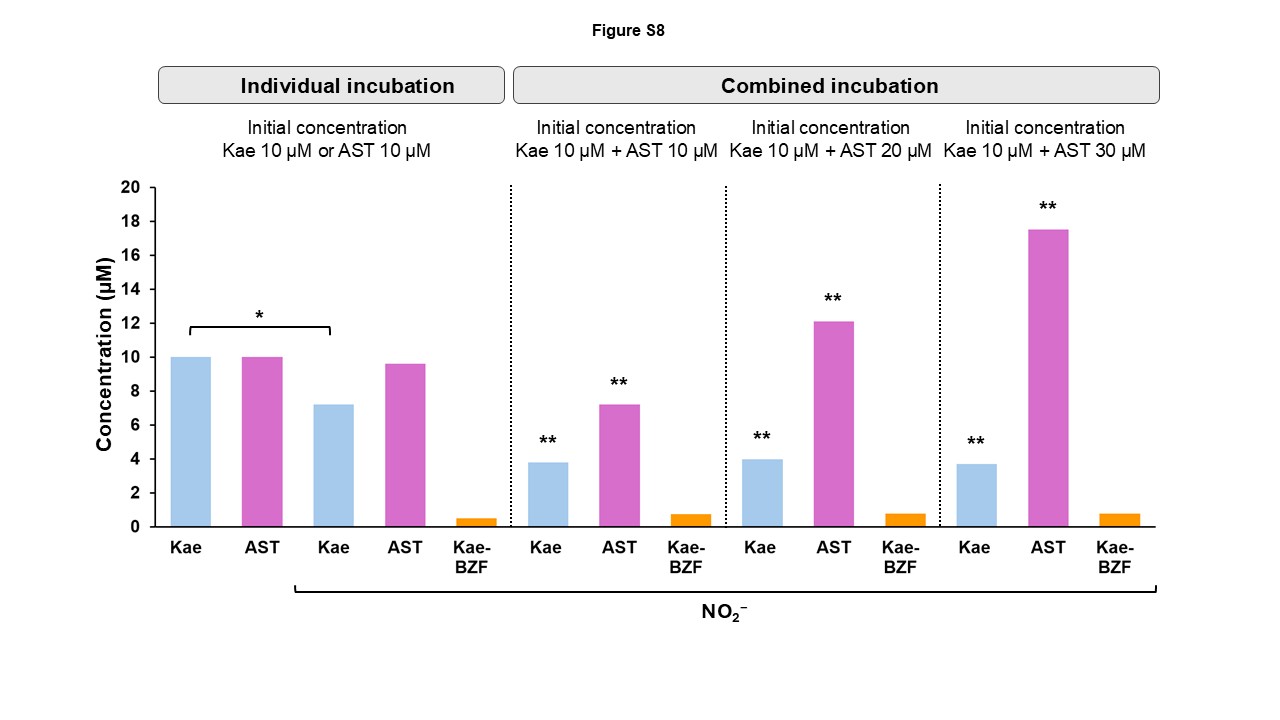

Supplement: Supplementary file 1 [file molecules-31-02320-s001.zip › Figure S8.jpg]

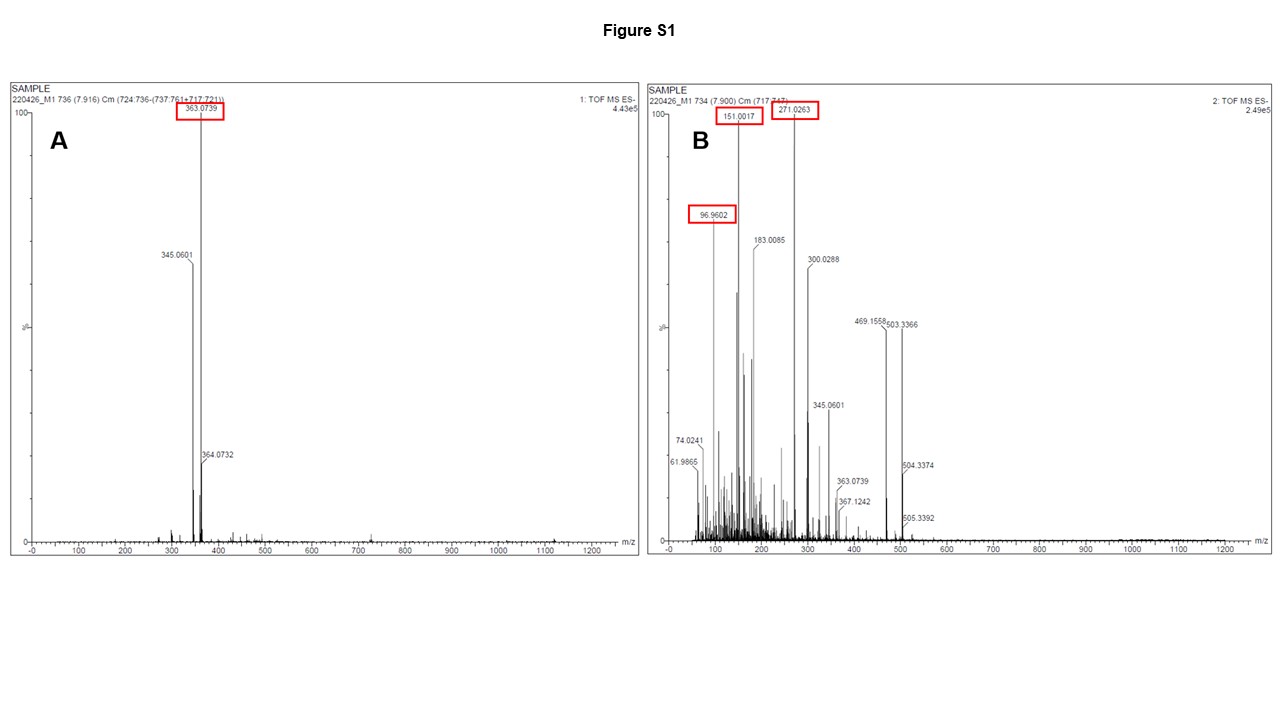

Supplement: Supplementary file 1 [file molecules-31-02320-s001.zip › Figure S1.jpg]

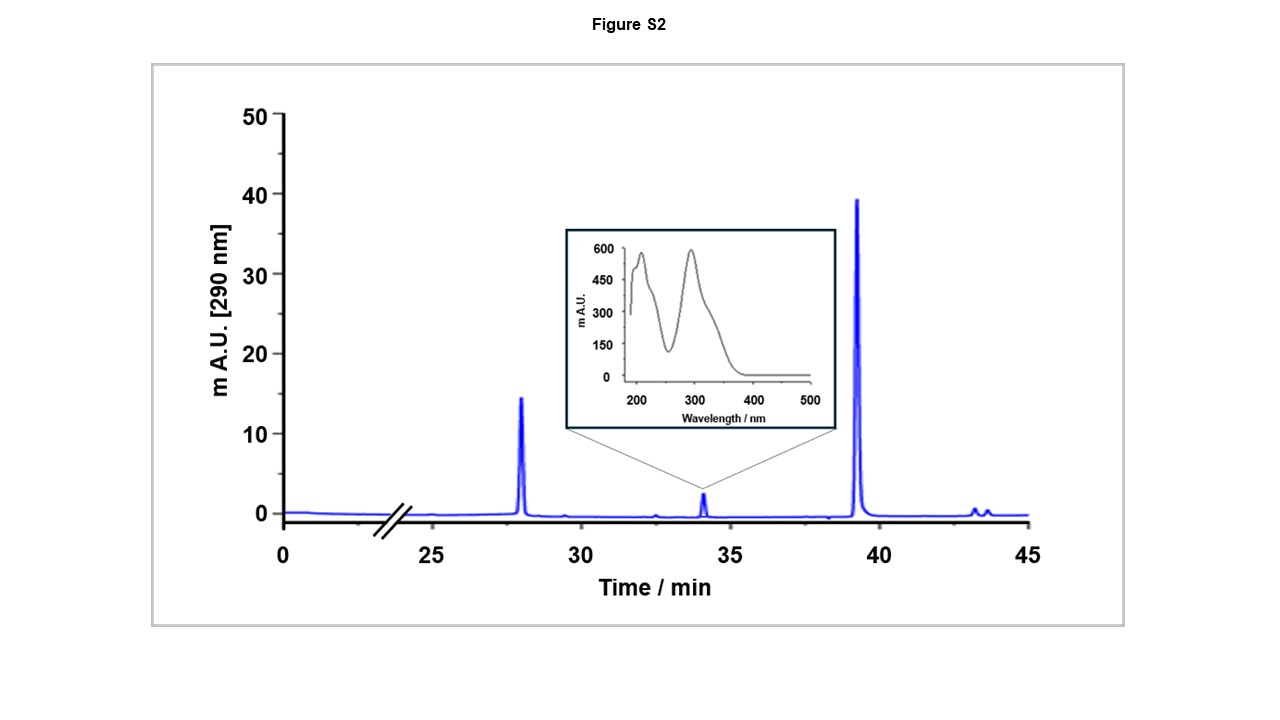

Supplement: Supplementary file 1 [file molecules-31-02320-s001.zip › Figure S2.jpg]

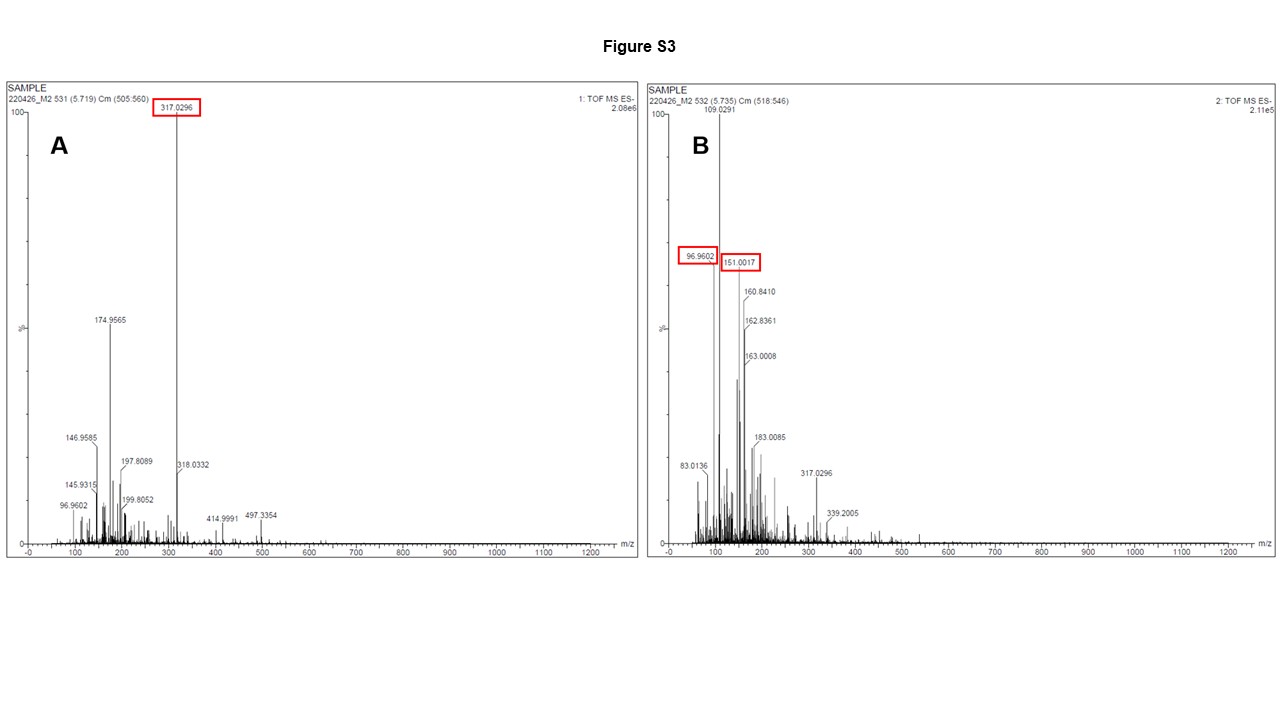

Supplement: Supplementary file 1 [file molecules-31-02320-s001.zip › Figure S3.jpg]

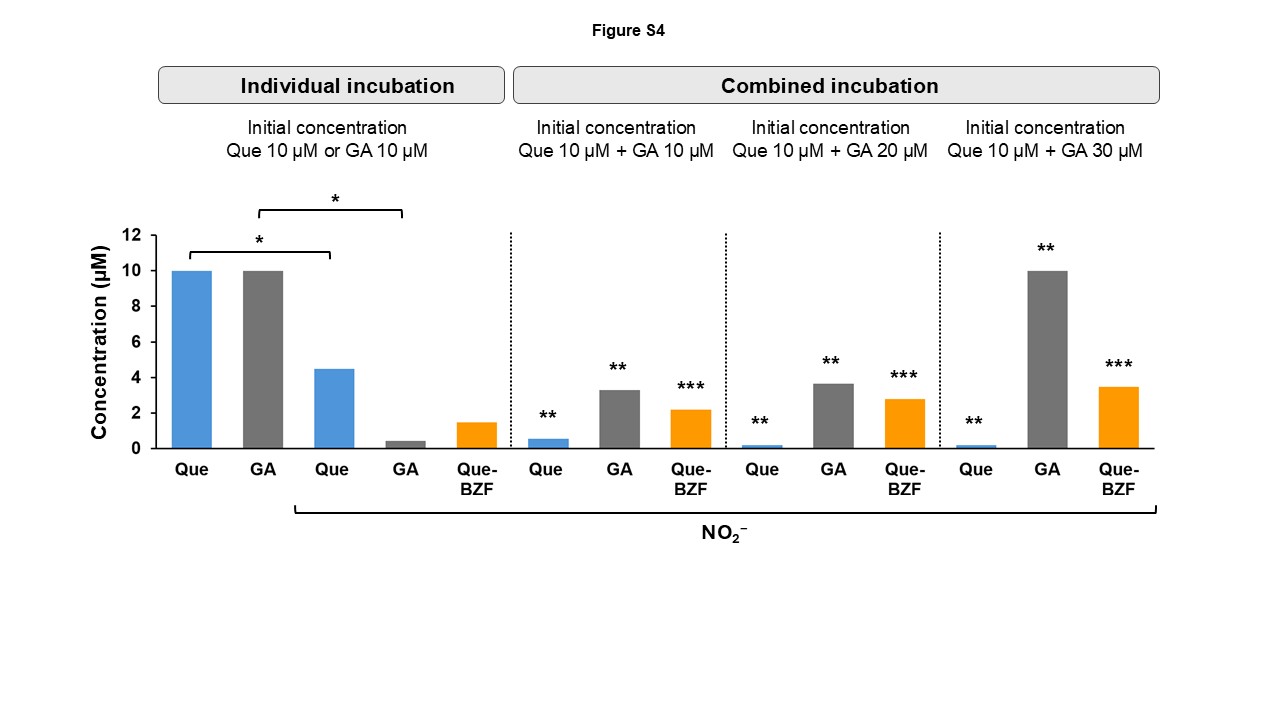

Supplement: Supplementary file 1 [file molecules-31-02320-s001.zip › Figure S4.jpg]

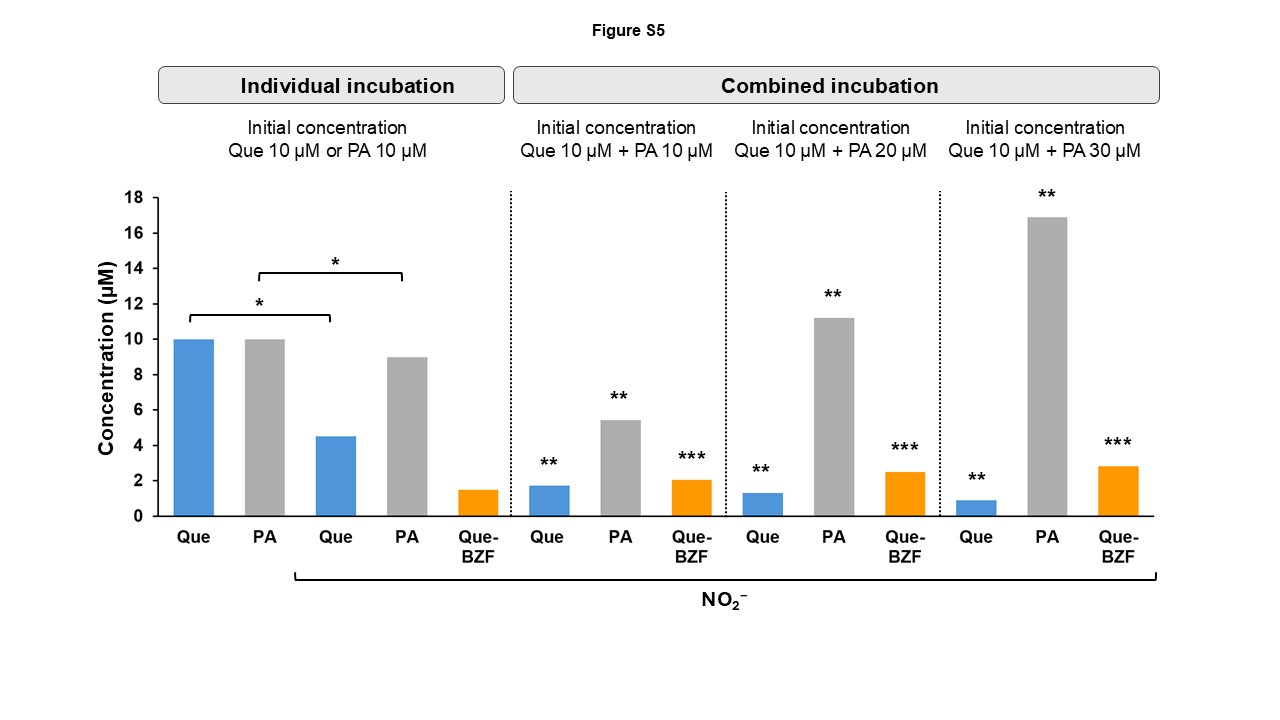

Supplement: Supplementary file 1 [file molecules-31-02320-s001.zip › Figure S5.jpg]

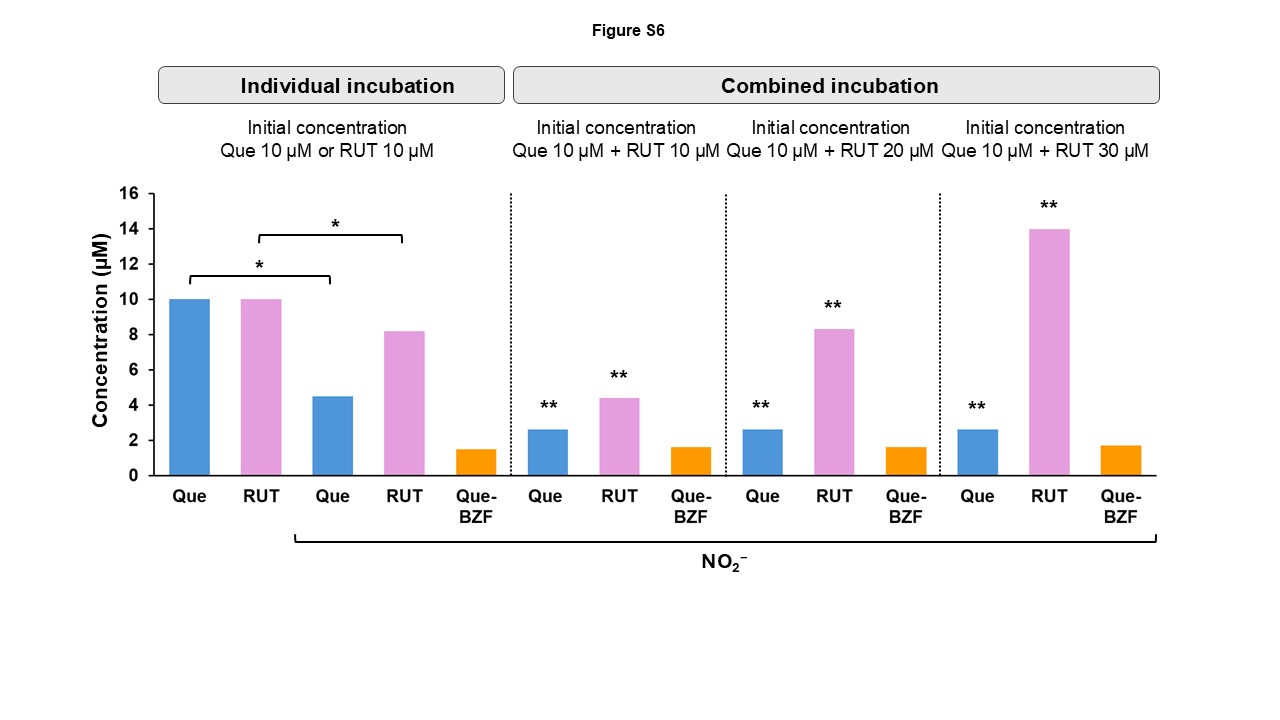

Supplement: Supplementary file 1 [file molecules-31-02320-s001.zip › Figure S6.jpg]

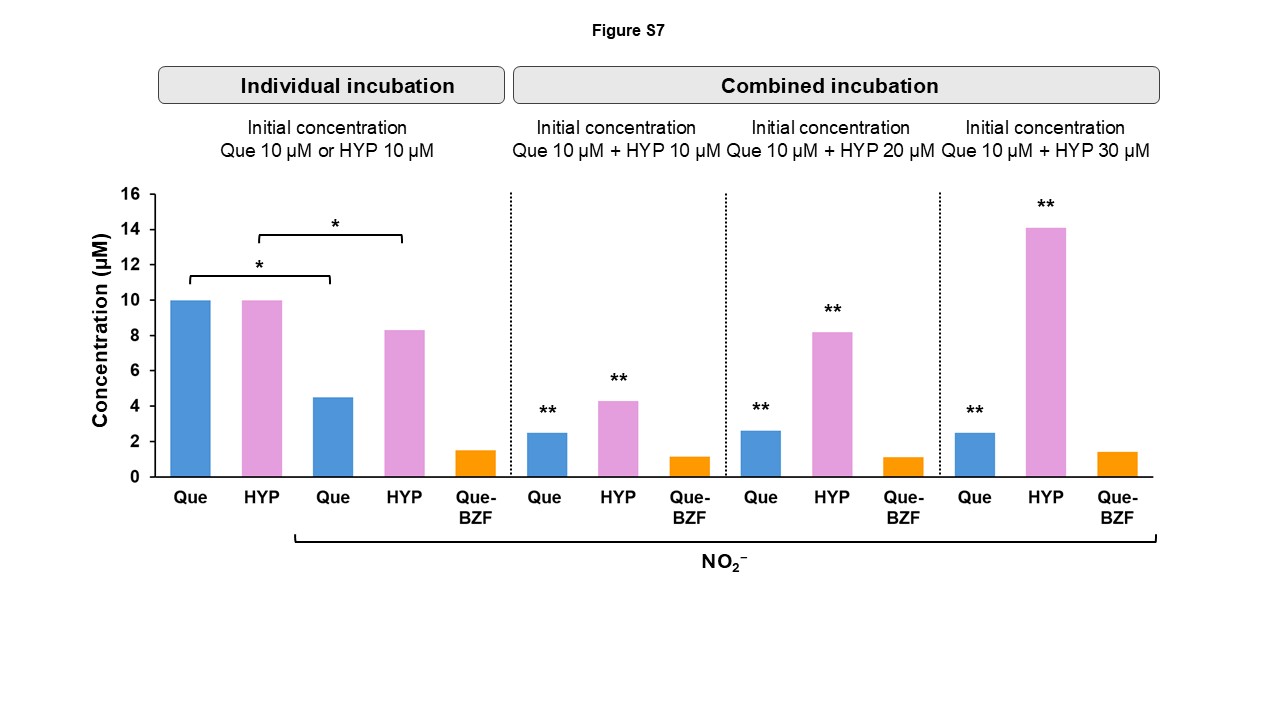

Supplement: Supplementary file 1 [file molecules-31-02320-s001.zip › Figure S7.jpg]
